# Snack food consumption among Bangladeshi children, supplementary data from a large RCT

**DOI:** 10.1111/mcn.12994

**Published:** 2020-03-20

**Authors:** Kaniz Jannat, Stephen P. Luby, Leanne Unicomb, Mahbubur Rahman, Peter J. Winch, Md. Iqbal Hossain, Christine P. Stewart

**Affiliations:** ^1^ School of Health Sciences Western Sydney University Sydney New South Wales Australia; ^2^ Division of Infectious Diseases and Geographic Medicine Stanford University Stanford California; ^3^ Infectious Disease Division and Nutrition and Clinical Services Division International Centre for Diarrhoeal Disease Research, Bangladesh Dhaka Bangladesh; ^4^ Department of International Health Johns Hopkins Bloomberg School of Public Health Baltimore Maryland; ^5^ Department of Nutrition University of California Davis Davis California

**Keywords:** dietary patterns, infant and child nutrition, infant feeding behaviour, infant feeding decisions, randomized controlled trial, water

## Abstract

Childhood obesity has been associated with consumption of energy‐dense foods such as caloric beverages and fast foods. Many low‐ and middle‐income countries like Bangladesh are now experiencing a rising problem of noncommunicable diseases along with the long‐standing problem of stunting and undernutrition. WASH Benefits Bangladesh was a large community‐based cluster randomized controlled trial conducted in rural Bangladesh. Study clusters were randomized into seven arms: single nutrition (N); water (W); sanitation (S); hygiene (H); combined water, sanitation, and hygiene (WSH); WSH and nutrition (N + WSH); and a double sized control (C). Nutrition intervention messages included four promotional components: maternal nutrition, breastfeeding, complementary feeding, and lipid‐based nutrient supplements. The World Health Organization infant food frequency questionnaire (24‐hr recall and 7‐day recall) was administered at Year 1 and Year 2 of intervention. The likelihood of any snack food consumption was significantly lower (odds ratio 0.37: 95% confidence interval [0.28, 0.49]) in the nutrition intervention arms compared to the control arm in Year 2 follow‐up. In addition, in the water intervention arm, fewer children (about 50% less) consumed soft drinks, but not the other sugar‐sweetened beverages, compared with control in Year 2. There were no other differences between groups. Simple messages about balanced diet and feeding family foods were effective in lowering commercially produced snack food consumption of the young children in low‐income rural communities of Bangladesh. Provision of safe water apparently encouraged mothers to reduce offering unhealthy beverages to the young children.

**Key messages**

- The promotion of optimal infant and young child feeding practices was associated with a passive reduction of commercially produced snack food consumption.
- Provision of safe water appeared to encourage mothers not to offer unhealthy beverages to young children.
- Snack food consumption was not associated with displacement of nutritious complementary foods; however, more research is needed using quantitative dietary intake assessment methods.

## 1 INTRODUCTION

A substantial worldwide increase in childhood obesity has posed a major global health challenge. The Global Burden of Disease Study 2013 identified that overweight and obesity among children have increased markedly in high‐, middle‐, and low‐income countries (Ng et al., 2014). Although the level of undernutrition continues to be high, some low‐ and middle‐income countries have experienced similar or greater increases in childhood obesity compared with high‐income countries (Lobstein et al., 2015). All dimensions of health, physical, emotional, and social, are profoundly influenced by childhood obesity (Sahoo et al., 2015). The World Health Assembly set a global nutrition target to halt the rise in childhood overweight by 2025 (WHO, 2013).

Childhood obesity has been associated with consumption of energy‐dense foods like caloric beverages and fast foods (Drewnowski & Specter, 2004). A study with more than 4,000 US‐children aged 5 and 11 years showed that there was an increased likelihood of developing overweight/obesity with increased frequency of fast food and sugar‐sweetened drink consumption (Berry, Burton, & Howlett, 2017). A longitudinal study on children of low socio‐economic status from Brazil also found that early ultraprocessed food consumption as a proportion of daily energy was associated with altered lipoprotein profile (Rauber, Campagnolo, Hoffman, & Vitolo, 2015); ultraprocessed foods are not made from whole foods but from their derivatives and food additives (Monteiro et al., 2012). Snacking habits such as consumption of sweet snacks, candy, and chips were associated with other adverse health outcomes including dental caries in young children (Johansson, Holgerson, Kressin, Nunn, & Tanner, 2010). Energy‐dense, nutrient‐poor foods have become more accessible to people at a lower cost due to developments in agriculture and food technologies (Drewnowski & Specter, 2004).

South Asian countries, including Bangladesh, have shown remarkable economic advancement in recent years. Improvement in household economic status, local production of packaged food, improved transportation, and extensive food marketing have bought changes in dietary patterns even to rural populations (Bishwajit, 2015). Up to 75% of 2‐year‐old Asian children from countries like Philippines and Nepal were found to consume sugary snack foods (Huffman, Piwoz, Vosti, & Dewey, 2014). A study in rural Indonesia showed that snack food that included fast food, soft drinks, traditional snack foods, candies, and desserts, and modern snack foods consumption, in respect to total energy and nutrient intake, was associated with lower height‐for‐age *z*‐score among schoolchildren (Sekiyama, Roosita, & Ohtsuka, 2012). Countries like Bangladesh and India are now detecting increasing rates of noncommunicable diseases along with the long‐standing problem of stunting and undernutrition (Bishwajit, 2015). With a national stunting prevalence of 36% (BDHS, 2014), Bangladesh is simultaneously experiencing an increase in the prevalence of overweight (3.6 to 7.9% from 1998 to 2015) and obesity (2 to 9% from 2004 to 2015) over time (Biswas, Islam, Islam, Pervin, & Rawal, 2017).

Young children's eating behaviour is presumed to be greatly influenced by their parents (Steinsbekk, Belsky, & Wichstrøm, 2016). A parenting program in Bangladesh to address early childhood health, growth, and development found the intervention was effective in changing mother's practices related to dietary diversity (Aboud, Singla, Nahil, & Borisova, 2013). A recent systematic review on the factors determining eating behaviour among preschool children in low‐ and middle‐income countries found that better nutritional knowledge of caregivers was associated with an increased healthy eating practices of the children (Sirasa, Mitchell, Rigby, & Harris, 2019). Therefore, an intervention focused on improved caregiver knowledge of appropriate complementary feeding practice may result in improved dietary quality and less unhealthy snack food consumption in their children.

The primary objective of this study was to measure commercially available snack food and packaged food consumption patterns among children less than 3 years of age participating since birth in a randomized controlled trial of nutrition, water, sanitation, and hygiene interventions. We previously reported a significant increase in dietary diversity among the children in the nutrition intervention group compared with control (Jannat et al., 2018). Thus, we were interested to explore if there were any relationships between snack food consumption and complementary food diversity among the children who took part in the intervention trial. We hypothesized that a nutrition intervention promoting diverse complementary foods with a focus on increasing fruits and vegetables, meat, fish, eggs, and dairy foods would reduce commercially available snack food consumption among the participating young children.

## 2 METHODS

### 2.1 Study design

WASH Benefits Bangladesh (clinicaltrials.gov identifier: NCC01590095) was a large community‐based cluster randomized control trial conducted in rural Bangladesh. The study sites included four central districts: Gazipur, Kishoreganj, Mymensingh, and Tangail. These sites were selected according to the quality of ground water and presence or absence of ongoing or upcoming water, sanitation, and nutrition interventions. At the initial stage of the study, research assistants conducted a community census to identify pregnant women. Women at their first or second trimester of pregnancy were eligible to enrol in the study. Eight nearest pregnant women, identified using a global positioning system, formed a study cluster. These study clusters were randomized into seven arms: single nutrition (N); water (W); sanitation (S); hygiene (H); combined water, sanitation, and hygiene (WSH); combined WSH and nutrition (N + WSH); and a double sized control (C). Each intervention arm contained 90 clusters, and the control arm contained 180 clusters. A total of 5,551 women and their offspring were enrolled in the study. The randomized group assignment could not be blinded due to the nature of the interventions. Detailed study design and rationale have been published previously (Arnold et al., 2013; Jannat et al., 2018; Luby et al., 2018).

### 2.2 Intervention delivery

The WASH Benefits Bangladesh study delivered household‐level interventions. The Integrated Behavioral Model for Water Sanitation and Hygiene (Dreibelbis et al., 2013) was used to develop the water, sanitation, and handwashing interventions. The water, sanitation, and handwashing interventions included behaviour change messages and low‐cost hardware to provide a supporting environment to practice the promoted behaviours. The nutrition intervention component adapted behaviour change recommendations developed by the Alive and Thrive programme in Bangladesh (Menon et al., 2016) and delivered small quantity lipid‐based nutrient supplements to children from 6 to 24 months of age. To deliver the intervention to the households, especially to the mothers, women from the local community were recruited as community health promoters (CHPs). CHPs were under regular supervision of the research assistants. Supervision of the research staffs were arranged in a ranked order so that the implementation remained even and precise. Supervisors were trained in direct intervention delivery; subsequently, they trained the CHPs. First round of training was more intensive including basic project description, principals of behaviour change, communication skills, intervention specific training, and role play. This was followed by regular monthly meeting between supervisors and CHPs. Refresher training was also arranged about a year after initiation of intervention (Unicomb et al., 2018). Each cluster of 6–8 months was monitored by one CHP. On average, a cluster was about 1 km in diameter that could easily be covered by a CHP. We maintained a 1 km of buffer zone in between the study clusters to prevent possible spillover effects.

Nutrition intervention messages had four promotional components: maternal nutrition, breastfeeding, complementary feeding, and provision of lipid‐based nutrient supplements. The intervention started at about the second trimester of pregnancy and continued up to the end of second year of child age. During pregnancy, mother's nutrition and regular antenatal check‐up were promoted. Exclusive breastfeeding counselling was initiated at the last trimester of pregnancy. Mothers who experienced challenges initiating exclusive breastfeeding were attended with special sessions. Information regarding balanced diet, amount, and frequency of complementary feeding was communicated to the mothers using illustrative flip charts. Recommendations for complementary feeding included providing two to four main meals according to child age and one to two healthy snacks a day. Healthy snacks referred to locally available fruits like banana and papaya and homemade snack items such as fried vegetables (pumpkin, green banana, and potato), rice pudding, and homemade cakes made with milk. Avoiding commercially produced unhealthy snack food consumption was not specifically included in the regular messaging. Offering unhealthy snack foods was discouraged only when mothers experienced difficulties establishing appropriate complementary feeding or feeding lipid‐based nutrient supplements. In this trial, we considered healthy snack items to be those that were minimally processed, such as those made from fresh ingredients or prepared at home. Homemade snacks are healthier options for rural children compared with available cheap commercial snack foods considering their amount of nutrient content, chemical contamination, and hygiene (Khairuzzaman, Chowdhury, Zaman, Al Mamun, & Bari, 2014). Mothers were encouraged to feed family foods that principally included rice and curry, to the children. To encourage appropriate timing of introduction to complementary foods, the day was marked with an event called *Mukhe vaat* (child eating rice for the first time). Reminder cue cards regarding balanced diet were distributed to the households. Lipid‐based nutrient supplements were distributed to the mothers on a monthly basis. Two 10‐g sachets were recommended for daily consumption starting from 6 months until 2 years of age.

### 2.3 Data collection

During the baseline assessment, before the index children were born, research assistants visited participants in their home to collect household socio‐economic and demographic data. The research assistants conducted two follow‐up visits at about 1 year and 2 years of commencement of intervention delivery. The infant food frequency questionnaire (24‐hr recall and 7‐days recall) was completed at both follow‐up visits. The infant and young child feeding module was adapted from indicators recommended by the World Health Organization (WHO, 2010a) with an added module to assess consumption of commercially produced snack and packaged foods. Commercially produced snacks were given special consideration due to their high salt, sugar, and trans‐fat content, and also taking into account the presence of food substances that are not commonly used in culinary preparations such as hydrogenated oil and food additives like colouring agents, flavourings, and nonsugar sweeteners (Araújo, Ribeiro, Padrão, & Moreira, 2019). We grouped different commonly available snack foods and packaged foods in the rural market according to their taste and pattern. The questionnaire module focused on store‐bought sweet and savoury snacks rather than those that were home prepared. Store‐bought foods may be ultraprocessed, contain high amounts of sugar, high sodium, or are deep fried with frying oil used and reheated several times, which increases the trans‐fat content of the food (Bhardwaj et al., 2016). We grouped them into six categories: soft drinks, fruit juice, sweetened milk and milk products, sweet snacks, savoury snacks, and pickles. Soft drinks included all variety of soda water available in the market such as Coca Cola, 7 Up, and Fanta. Fruit juices that were packaged commercially, not freshly prepared, were included in the fruit juice category such as artificial orange juice, mango juice, and lychee drinks. The bottled and canned milk category referred to milk with added sugar, colour, flavour, or chocolate. Milk products, which are made commercially, with added sugar, colour, flavour, or chocolate such as ice cream and other locally available products (*Kulfi*) were classified in milk product category. Sweet snacks included chocolate, candy, and sweet meats prepared commercially. Savoury snacks were deep‐fried snacks with added sodium and trans‐fats, like chips and *chanachur* (a locally available deep‐fried snack). Pickles included fruits (vegetables are not usually preserved as pickle in Bangladesh) preserved with salt and oil. The research assistants asked mothers about each type of snack food whether it was consumed by the index children within the past 7 days. If any item from the mentioned food categories was consumed, mothers were asked for how many days the particular food item was consumed in past 7 days.

### 2.4 Data analysis

The outcomes presented in this paper were added during the course of the study and were not considered primary or secondary outcomes of the trial. Those results have been published elsewhere (Luby et al., 2018; Tofail et al., 2018). Nevertheless, the statistical analyses were planned and pre‐specified prior to beginning the analysis. The WHO has defined snack foods for young children as foods that are easy to prepare, mostly self‐fed, and eaten in between main meals (WHO, 2005). In this paper, we defined snack food as unhealthy when they contained excess sugar (these included as soft drinks, artificial fruit juice, sweetened milk, and sweet snacks), high salt (included as savoury snacks such as chips), trans‐fat, and/or low in nutritional value (WHO, 2010b). Individual proportions of the six categories of snack food consumption (at least once in past 7 days) were estimated for children from each of the intervention arms and controls. Then, we combined all the individual proportions to measure child consumption of any of these six listed snack foods groups at least once in past 7 days. All these proportions of consumption were compared between interventions and controls. Minimum dietary diversity was adapted from the WHO indicator (WHO, 2010a) and classified as a binary variable based on whether children consumed foods from ≥4 groups among seven recommended food groups all days within past 7 days. We used a multivariate logistic regression model to measure if there was any association between snack food consumption and minimum dietary diversity in the past 7 days. We added the covariates: child age and sex, parent's education, father's occupation, study districts, household socio‐economic status, and food insecurity in the model. This model was used to assess the association among children from the nutrition or nonnutrition arms and controls separately. We examined that the association between snack food consumption and dietary diversity in the nutrition arm and other arms as dietary diversity was similar among children from control and nonnutrition arms (Jannat et al., 2018). Food insecurity was measured using WHO Household Food Insecurity Access Scale (Coates, Swindale, & Bilinsky, 2007). Because participating children were randomized at cluster level, it was expected that responses were correlated within cluster. In order to obtain cluster‐robust standard errors, all analyses were adjusted for clustering using the sandwich estimator (Carroll, Wang, Simpson, Stromberg, & Ruppert, 1998).

### 2.5 Ethical considerations

At the time of enrolment, written informed consent was obtained from the household head, the pregnant women, and guardians of the children <36 months of age. The study procedures, what was expected from the study participants, risk and benefits of participating in the research study, and the rights for withdrawal were explained to the participants. Randomization procedure was simplified to the participants for better understanding. The study was approved by the icddr,b (PR‐11063), the University of California Berkeley (2011‐09‐3652), and the Stanford University (25863) institutional review boards.

## 3 RESULTS

**FIGURE 1:**
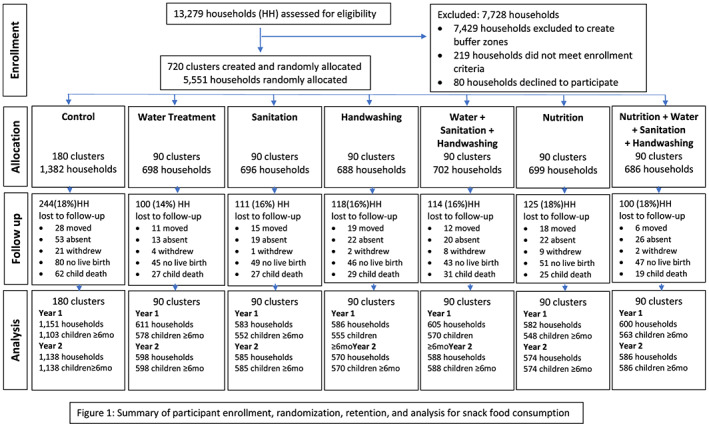
Summary of participant enrolment, randomization, retention, and analysis for snack food consumption

Among the 5,551 households included in the study, the research assistants interviewed 4,716 mothers at Year 1 follow‐up and 4,639 mothers at Year 2 follow‐up visits (Figure 1). The mothers provided information regarding complementary feeding and snack foods consumption when children were at a mean age of 8.7 months (1.7 standard deviation [*SD*]) at Year 1 and 22.0 months (2.0 *SD*) at Year 2.

Baseline characteristics, child age, and sex were balanced across study arms (Table 1). About half of the mothers had an educational level of more than grade five. Electricity connection was available in around 60% of the households. The household size was around five persons (2 *SD*) per family. Food insecurity was reported by only around a quarter (30%) of the households.

**TABLE 1:** Characteristics of the study population at baseline and children at follow‐up

Indicators	Control^a^	Water	Sanitation	Handwashing	WSH	Nutrition	N + WSH
	*N* = 1382 % (95% CI)	*N* = 698 % (95% CI)	*N* = 696 % (95% CI)	*N* = 688 % (95% CI)	*N* = 702 % (95% CI)	*N* = 699 % (95% CI)	*N* = 686 % (95% CI)
Household characteristics							
Has electricity	57 (54, 59)	60 (57, 64)	59 (55, 62)	59 (55, 63)	61 (57, 64)	59 (55, 62)	60 (56, 64)
Household size (mean, SD)	4.7 (2.3)	4.6 (2.2)	4.7 (2.1)	4.7 (2.2)	4.7 (2.1)	4.7 (2.2)	4.7 (2.1)
Food insecure^b^	33 (30, 35)	29 (26, 33)	32 (28, 35)	31 (28, 35)	31 (28, 35)	31 (28, 35)	29 (26, 33)
Paternal characteristics							
Occupation of the father							
Agriculture	30 (28, 32)	32 (29, 36)	29 (26, 33)	36 (33, 40)	31 (27, 34)	33 (30, 37)	30 (27, 34)
Service or business	30 (27, 32)	27 (24, 30)	30 (27, 34)	28 (24, 31)	31 (28, 34)	31 (27, 34)	31 (28, 35)
Working abroad	7 (5,8)	7 (5,9)	7 (5,9)	5 (3,7)	6 (4,8)	6 (4,8)	5 (4,7)
Other	34 (31, 36)	35 (31, 38)	34 (31, 38)	31 (28, 35)	32 (29, 36)	30 (27, 34)	34 (30, 37)
Education, more than primary	41 (38, 43)	40 (36, 43)	41 (37, 44)	37 (34, 41)	40 (37, 44)	40 (36, 43)	37 (34, 41)
Maternal characteristics							
Education, more than primary	53 (51, 56)	54 (50, 58)	52 (48, 56)	53 (49, 57)	54 (50, 58)	54 (50, 57)	50 (46, 54)
Diet in past 7 days (≥1 day)							
Meat, fish, or eggs	99 (99, 100)	100	100	100	99 (99, 100)	99 (98, 100)	99 (99, 100)
Dairy	53 (50, 55)	54 (50, 58)	54 (51, 58)	54 (50, 58)	51 (48, 55)	50 (46, 54)	55 (51, 58)
Vitamin A rich (yellow) fruits or veg.	47 (45, 50)	48 (44, 52)	48 (44, 52)	47 (43, 50)	48 (44, 51)	50 (46, 53)	50 (46, 54)
Child characteristics at follow‐up							
Sex (male) at Year 1	49 (46, 52)	50 (46, 54)	51 (47, 55)	50 (46, 55)	52 (48, 56)	51 (47, 55)	47 (43, 51)
Sex (male) at Year 2	50 (47, 53)	50 (46, 54)	51 (46, 55)	49 (45, 53)	52 (48, 56)	51 (47, 55)	46 (42, 50)
Age (mo) at Year 1 (mean, *SD*)	8.8 (1.7)	8.8 (1.7)	8.8 (1.7)	8.8 (1.7)	8.7 (1.8)	8.6 (1.7)	8.7 (1.7)
Age (mo) at Year 2 (mean, *SD*)	22.4 (2.0)	22.5 (2.0)	22.5 (2.0)	22.5 (2.1)	22.4 (2.1)	22.4 (2.1)	22.4 (2.0)

Abbreviations: CI, confidence interval; OR, odds ratio; *SD*, standard deviation; WSH, water, sanitation and hygiene; WSHN, WSH and nutrition.

a None of the indicators were significantly different compared to the control arm.

b Food insecurity was measured using World Health Organization Household Food Insecurity Access Scale.

The prevalence of snack food consumption in past 7 days in different arms was 0–7% at the Year 1 follow‐up. This prevalence increased to 3–76% in Year 2 follow‐up (Table 2). Among all snack foods, consumption of sweet snacks (867/1138, 76%) and savoury snacks (807/1138, 71%) were highest in the control arm at Year 2. In the Year 2 follow‐up, consumption was significantly lower for soft drinks (N: 4%; WSHN: 3% vs. C: 7%), fruit juice (N: 13%; WSHN: 14% vs. C: 21%), sweet snacks (N: 58%; WSHN: 56% vs. C: 76%), savoury snacks (N: 51%; WSHN: 47% vs. C: 71%), and pickles (N: 11%; WSHN: 11% vs. C: 17%). Consumption of sweetened milk and milk products was significantly lower in the single nutrition arm but not in combined WSHN (N:10%; WSHN: 14% vs. C: 15%). In addition, consumption of soft drinks was lower (W: 4% vs. C: 7%) in the water intervention arm compared with control in Year 2 (Table 2). The consumption of any snack food at least once in the past 7 days was significantly lower (N: 73%; N + WSH: 69% vs. C: 87%) in the nutrition intervention arms compared with the control arm at Year 2 follow‐up (Table 3).

**TABLE 2:** Effect of the nutrition intervention on snack and packaged food consumption (at least once in last 7 days)

	Year 1				Year 2			
	*N*	% (95% CI)	OR	95% CI	*N*	% (95% CI)	OR	95% CI
Soft drinks								
Control	1,103	0.5 [0.2, 1.2]	Ref	Ref	1,138	6.6 [5.2, 8.2]	Ref	Ref
Water	578	0.7 [0.2, 1.8]	1.27	0.33, 4.86	598	3.8 [2.5, 5.7]	**0.57**	**0.35, 0.92**
Sanitation	552	0.5 [0.1, 1.6]	1.00	0.23, 4.25	585	6.5 [4.6, 8.8]	0.98	0.60, 1.61
Handwashing	555	0.4 [0, 1.3]	0.66	0.08, 5.78	570	5.0 [3.3, 7.0]	0.73	0.43, 1.24
WSH	570	1.4 [0.6, 2.7]	2.60	0.84, 8.11	588	7.0 [5.0, 9.3]	1.06	0.67, 1.68
Nutrition	548	0.4 [0, 1.3]	0.67	0.13, 3.50	574	3.7 [2.3, 5.5]	**0.54**	**0.33, 0.89**
WSHN	563	0.2 [0, 1.0]	0.33	0.04, 2.82	586	2.7 [1.6, 4.4]	**0.40**	**0.24, 0.67**
Canned fruit juice								
Control	1,103	5.2 [4.0, 6.6]	Ref	Ref	1,138	21.4 [19.0, 24.0]	Ref	Ref
Water	578	5.5 [3.8, 7.7]	1.08	0.75, 1.79	598	20.7 [17.6, 24.2]	0.96	0.71, 1.31
Sanitation	552	6.2 [4.3, 8.5]	1.20	0.75, 1.94	585	22.0 [18.6, 25.5]	1.03	0.78, 1.37
Handwashing	555	6.5 [4.6, 8.9]	1.27	0.77, 2.12	570	19.6 [16.5, 23.2]	0.90	0.67, 1.22
WSH	570	6.5 [4.6, 8.8]	1.27	0.75, 2.15	588	21.3 [18.0, 24.8]	1.00	0.72, 1.37
Nutrition	548	5.7 [3.9, 8.0]	1.10	0.64, 1.91	574	13.0 [10.3, 16.0]	**0.55**	**0.39, 0.77**
WSHN	563	4.1 [2.6, 6.1]	0.78	0.44, 1.38	586	14.0 [11.3, 17.1]	**0.60**	**0.43, 0.83**
Sweetened milk and milk products								
Control	1,103	0.5 [0.1, 1.1]	Ref	Ref	1,138	14.9 [12.8, 17.1]	Ref	Ref
Water	578	0	‐	‐	598	13.4 [10.8, 16.4]	0.89	0.60, 1.31
Sanitation	552	0.5 [0.1, 1.6]	1.2	0.29, 4.95	585	14.0 [11.3, 17.1]	0.93	0.65, 1.34
Handwashing	555	0.4 [0, 1.3]	0.79	0.09, 6.73	570	14.4 [11.6, 17.5]	0.96	0.67, 1.39
WSH	570	0.5 [0.1, 1.5]	1.16	0.28, 4.77	588	15.5 [12.6, 18.7]	1.05	0.71, 1.56
Nutrition	548	0	‐	‐	574	9.8 [7.5, 12.5]	**0.62**	**0.40, 0.96**
WSHN	563	0.2 [0, 1.0]	0.39	0.05, 3.33	586	13.8 [11.1, 17.0]	0.92	0.62, 1.36
Sweet snacks/candy								
Control	1,103	5.0 [3.8, 6.4]	Ref	Ref	1,138	76.2 [73.6, 78.6]	Ref	Ref
Water	578	7.4 [5.4, 9.9]	**1.53**	**1.00, 2.37**	598	72.4 [68.6, 76.0]	0.82	0.64, 1.05
Sanitation	552	3.6 [2.2, 5.5]	0.72	0.41, 1.25	585	74.0 [70.3, 77.5]	0.89	0.68, 1.16
Handwashing	555	4.0 [2.5, 6.0]	0.79	0.47, 1.33	570	69.3 [65.3, 73.1]	**0.71**	**0.54, 0.92**
WSH	570	6.5 [4.6, 8.8]	1.32	0.80, 2.19	588	76.0 [72.4, 79.4]	1.00	0.76, 1.30
Nutrition	548	3.8 [2.4, 5.8]	0.76	0.45, 1.29	574	57.5 [53.3, 61.6]	**0.42**	**0.33, 0.55**
WSHN	563	3.0 [1.8, 4.8]	0.59	0.33, 1.07	586	56.3 [52.2, 60.4]	**0.40**	**0.31, 0.52**
Savoury snacks								
Control	1,103	1.8 [1.1, 2.8]	Ref	Ref	1,138	70.9 [68.2, 73.5]	Ref	Ref
Water	578	4.7 [3.1, 6.7]	**2.65**	**1.45, 4.86**	598	65.6 [61.6, 69.4]	0.78	0.62, 1.00
Sanitation	552	3.4 [2.1, 5.3]	1.93	0.98, 3.80	585	67.5 [63.6, 71.3]	0.85	0.67, 1.08
Handwashing	555	3.2 [2.0, 5.1]	1.82	0.96, 3.43	570	66.3 [62.3, 70.2]	0.81	0.63, 1.04
WSH	570	2.8 [1.6, 4.5]	1.56	0.81, 3.03	588	67.9 [64.0, 71.6]	0.87	0.68, 1.10
Nutrition	548	0.9 [0.3, 2.1]	0.50	0.19, 1.31	574	50.5 [46.4, 54.7]	**0.42**	**0.33, 0.52**
WSHN	563	0.7 [0.2, 1.8]	0.39	0.13, 1.12	586	46.6 [42.5, 50.7]	**0.36**	**0.28, 0.46**
Pickles								
Control	1,103	0.4 [0.1, 1.0]	Ref	Ref	1,138	17.0 [14.8, 19.3]	Ref	Ref
Water	578	0.9 [0.3, 2.0]	2.40	0.58, 9.87	598	19.0 [15.8, 22.3]	1.14	0.87, 1.50
Sanitation	552	0.7 [0.2, 1.8]	2.00	0.51, 7.91	585	16.6 [13.7, 19.8]	0.98	0.73, 1.29
Handwashing	555	0.4 [0, 1.3]	1.00	0.18, 5.37	570	14.7 [12.0, 18.0]	0.85	0.62, 1.16
WSH	570	0.5 [0.1, 1.5]	1.45	0.33, 6.46	588	19.0 [16.0, 22.5]	1.15	0.90, 1.48
Nutrition	548	0.4 [0, 1.3]	1.01	0.19, 5.44	574	10.8 [8.4, 13.6]	**0.59**	**0.42, 0.83**
WSHN	563	0.9 [0.3, 2.1]	2.46	0.67, 9.08	586	10.6 [8.2, 13.4]	**0.58**	**0.40, 0.82**

*Note.* Statistically significant differences are in bold fonts.

Abbreviations: CI, confidence interval; OR, odds ratio; WSH, water, sanitation, and hygiene; WSHN, WSH and nutrition.

**TABLE 3:** Consumption of any snack food or packaged food in past 7 days across arms

	Year 1				Year 2			
	*N*	% (95% CI)	OR	95% CI	*N*	% (95% CI)	OR	95% CI
Any snack food consumed at least once in past 7 days								
Control	1,103	11.1 [9.3, 13.1]	Ref	Ref	1,138	87.4 [85.4, 89.3]	Ref	Ref
Water	578	15.4 [12.6, 18.6]	**1.46**	**1.03, 2.09**	598	86.5 [83.4, 89.1]	0.90	0.65, 1.24
Sanitation	552	12.7 [10.0, 15.7]	1.17	0.81, 1.68	585	85.1 [82.0, 88.0]	0.81	0.60, 1.09
Handwashing	555	11.5 [9.0, 14.5]	1.05	0.72, 1.54	570	84.0 [80.8, 87.0]	0.74	0.55, 1.01
WSH	570	13.5 [10.8, 16.6]	1.26	0.86, 1.83	588	89.0 [86.1, 91.4]	1.13	0.80, 1.60
Nutrition	548	10.4 [8.0, 13.3]	0.93	0.61, 1.43	574	72.6 [68.8, 76.3]	**0.37**	**0.28, 0.49**
WSHN	563	7.8 [5.7, 10.3]	0.68	0.45, 1.04	586	69.3 [65.4, 73.0]	**0.31**	**0.23, 0.41**

*Note.* Statistically significant differences are in bold fonts.

Abbreviations: CI, confidence interval; OR, odds ratio; WSH, water, sanitation, and hygiene; WSHN, WSH and nutrition.

Some of the snack foods consumption was associated with increased dietary diversity (Table 4). Specifically, among the control and nonnutrition arms, canned fruit juices consumption was associated with a higher odds of dietary diversity at Year 2 (Table 4). In the nutrition arms at Year 2 follow‐up, consumption of soft drinks, sweet snacks, and pickles was associated with an increased consumption of diverse complementary foods (Table 4). In addition, when measuring association between snack food consumption and dietary diversity, higher educational attainment of fathers was consistently associated with an increased food diversity**.**

**TABLE 4:** Association between snack food consumption and dietary diversity among children

Snack food consumption in past 7 days	Dietary diversity (consumption of ≥4 food groups all days in past 7 days)			
	Year 1		Year 2	
	Control + other intervention	Nutrition intervention	Control + other intervention	Nutrition intervention
	OR^a^ (95% CI)	OR^a^ (95% CI)	OR^a^ (95% CI)	OR^a^ (95% CI)
Soft drinks	1.22 (0.42, 3.57)	5.86 (0.65, 53.09)	1.31 [0.97, 1.76]	**2.14 [1.03, 4.42]**
Canned fruit juice	1.46 (0.96, 2.21)	1.27 (0.77, 2.12)	**1.27 [1.06, 1.53]**	1.05 [0.73, 1.50]
Sweetened milk and milk products	3.80 (0.89, 16.18)	‐	0.92 [0.74, 1.15]	1.08 [0.67, 1.74]
Sweet snacks/candy	0.84 (0.47, 1.52)	0.91 (0.44, 1.87)	0.99 [0.81, 1.20]	**1.45 [1.12, 1.86]**
Savoury snacks	1.06 (0.54, 2.07)	1.25 (0.25, 6.33)	1.19 [0.99, 1.43]	0.84 [0.64, 1.06]
Pickles	1.80 (0.50, 6.51)	1.05 (0.19, 5.66)	1.19 [0.97, 1.44]	**1.60 [1.05, 2.44]**

*Note.* Statistically significant differences are in bold fonts.

a Results are adjusted for child age, sex, parents' education, fathers' occupation, study districts, household wealth, and food insecurity.

## 4 DISCUSSION

In this randomized controlled trial of nutrition, water, sanitation, and hygiene, children participating in the nutrition intervention were less likely to consume sweets or packaged snack food items. Snack food consumption was significantly lower in the nutrition intervention arms compared with the control arm, particularly in the Year 2 follow‐up. Although commercial snack food consumption was not actively discouraged, promotion of healthy complementary foods lowered commercial snack foods intake by up to 50% among children who participated in the nutrition intervention. In addition, mothers in the water intervention arm, who received a water‐purifying agent and a safe water storage container, were less likely to offer their children unhealthy drinks like soft drinks. We found that consumption of snack or packaged foods was associated with a higher likelihood of children meeting the minimum dietary diversity score.

This passive influence of the nutrition and water interventions on snack foods and drinks consumption provided us with evidence that a well‐designed and well implemented intervention may have the potential to influence behaviours beyond just those explicitly targeted. WASH Benefit trial used the Integrated Model for Water Sanitation and Hygiene that was developed over 2 years of field evaluation. The model took account into contextual, psychological, and technological factors at different levels starting from individual to societal level (Dreibelbis et al., 2013).

We previously reported that the nutrition intervention, which focused on the timely introduction of diverse complementary foods, resulted in a higher dietary diversity score at both time points (Jannat et al., 2018) and high adherence to lipid‐based nutrient supplementation recommendations (Luby et al., 2018). The present data suggest that parents prioritized feeding the promoted nutritious foods or lipid‐based nutrient supplements over commercially available sweet, salty, or packaged snack foods to their children. Parents have more control over children's food environment and experiences at an early age (Anzman, Rollins, & Birch, 2010). Similar to the WASH Benefits intervention, studies that delivered interventions targeting the parents were found to be effective in improving children's dietary behaviour (Kader, Sundblom, & Elinder, 2015). The Alive and Thrive program in Bangladesh found that minimum dietary diversity improved by 16.3 percentage points among the children who were in intensive intervention group compared to those in the nonintensive group (Menon et al., 2016). Interventions targeting parent's behaviour towards children's snacking habits is scarce in Southeast Asia; however, there is a clear and growing need to address this rising problem. A cross‐sectional survey conducted in the Kathmandu valley of Nepal revealed that poor growth outcomes and inadequate micronutrient intakes were associated with a high unhealthy snack foods and beverages consumption (Pries et al., 2019). Another cross‐sectional survey in Nepal indicated a gap between the knowledge, attitude, and practice of the mothers regarding children's diet and physical activities (Oli, Vaidya, Pahkala, Eiben, & Krettek, 2018).

The significant reduction in soft drinks in the water intervention arm is also notable. It is possible that when households felt that their own water was safer, they were less likely to feed their children with packaged drinks. Studies in the United States have also found that perceived water safety was associated with lower intake of sugar‐sweetened beverages (Onufrak, Park, Sharkey, Merlo, et al., 2014; Onufrak, Park, Sharkey, & Sherry, 2014). Future research should examine whether concerns about unsafe water might motivate families to drink bottled drinks in this context.

The proportion of snack food and packaged food consumption was very low (<10%) in the Year 1 follow‐up when children were less than 1 year of age. However, the consumption increased several folds, up to 75% for sweet or savoury snack foods by the end of the second year. A study by Huffman et al. on children 6–23 months of age from 18 Asian and African countries found that consumption of sugary snack foods increased with age (Huffman et al., 2014). The study also found that up to 75% of the Asian children consumed sugary snack foods in their second year of life. Similar findings were evident in developed countries like Finland, where sugary snack consumption was as high as 92% by 2 years of age (Laitala, Vehkalahti, & Virtanen, 2018). Consumption of energy dense foods and sugar‐sweetened drinks may result in an increased body mass index in children and adults (Collison et al., 2010; Pérez‐Escamilla et al., 2012). To regulate this practice of increased consumption, positive food parenting has an important role (Larsen et al., 2015). It is thus important to educate caregivers to support building healthy food habits among children.

We were interested to know how snack food consumption was associated with dietary diversity. We presumed that children who consumed more snack foods would have less diverse complementary foods. However, it was not apparent from our analysis that an increase in consumption of any particular type of snack food was related to a reduction in dietary diversity. In contrast, children who consumed more snack foods were relatively more likely to consume diverse foods, even after controlling for socioeconomic status, education, and other factors. In Year 2, children in the nonnutrition intervention arms who consumed canned fruit juices were more likely to meet the minimum dietary diversity score compared with those who did not consume canned fruit juices; in the nutrition intervention arm consumption of soft drinks, sweet snacks and pickles was associated with an increased dietary diversity. Although it was encouraging to see that snack food consumption was not reducing dietary diversity of complementary foods, these results should be interpreted with caution because calorie intake was not quantified. Nevertheless, the large increase in sweet and savoury snack consumption in the second year of life was concerning and might predispose children towards unhealthy snacking habits leading to overweight or obesity (St‐Onge, Keller, & Heymsfield, 2003). Studies have shown that adult food preferences take shape early in life (Schwartz, Scholtens, Lalanne, Weenen, & Nicklaus, 2011). Thus, promotion of healthy eating habits early in life is important to prevent obesity and subsequent metabolic dysfunctions.

The WASH Benefits Bangladesh trial was a large randomized control trial, which was designed to measure small differences in outcome variables, maintained high levels of implementation fidelity, and achieved high follow‐up rates throughout the 2‐year trial (Luby et al., 2018). Promoted behaviours showed a high uptake (Parvez et al., 2018), including an increase in dietary diversity in the nutrition intervention arm (Jannat et al., 2018). There were some limitations to this study that should be noted, however. Both complementary feeding and snack food consumption information were reported by the mothers. All food consumption was measured by frequency of intake not by quantity; thus, precise estimation of calories consumed was not possible. Therefore, we could not calculate snack food consumption as a percent of energy or directly estimate the possibility of displacement of healthy foods. Moreover, there might be inconsistencies in food categories with respect to their nutrient density. In the future, a nutrient profiling method would allow for a more standardized method for categorization of food types (Drewnowski, Dwyer, King, & Weaver, 2019). Because the intervention was not blinded to either the participants or the data collectors, the chance of information bias cannot be ruled out. However, we used standardized dietary indicators to assess food diversity (WHO, 2010a). We adapted a new module for assessing commercially produced snack food consumption that covered most of the common snack items available in the rural community of Bangladesh. Rigorous training was conducted for the data collectors. The survey team worked independent of the intervention team to minimize surveyor's bias. Loss to follow‐up was reasonable (~15%) over a 2‐year period.

Simple messages about balanced diet and feeding family food were effective in lowering commercially produced snack food consumption of the young children in low‐income rural communities of Bangladesh. Provision of safe water appeared to encourage mothers to reduce offering unhealthy soft drinks to the children. Nevertheless, a sharp increase in snack food consumption in the second year of child age suggests that greater attention is needed to prevent the establishment of unhealthy eating behaviours in children.

## CONFLICTS OF INTEREST

The authors declare that they have no conflicts of interest.

## AUTHOR CONTRIBUTIONS

KJ drafted the manuscript under the guidance of CPS and input from all listed co‐authors. SPL drafted the research protocol; he coordinated input from the study team throughout the project. PJW, MR, and LU developed the water, sanitation, and handwashing intervention and the overarching behaviour change strategies. CPS developed the nutrition intervention and guided the analysis and interpretation of these results. MR, LU, IH, and KJ oversaw piloting and subsequent study implementation, contributed to refinements in interventions and measurements, and responded to threats to validity. KJ and CPS developed the analytical approach, conducted the statistical analysis, constructed the tables and figures, and helped interpret the results.
